# Impact of Labor and Delivery Simulation Classes in Undergraduate Medical Learning

**DOI:** 10.3885/meo.2008.Res00285

**Published:** 2008-11-15

**Authors:** A Reynolds, D Ayres-de-Campos, LF Bastos, WL van Meurs, J Bernardes

**Affiliations:** * Departamento de Ginecologia e Obstetrícia, Centro de Simulação Biomédica, Faculdade de Medicina, Universidade do Porto, Portugal; † INEB-Instituto de Engenharia Biomédica, Divisão de Sinal e Imagem, Porto, Portugal; ‡ Department of Anesthesiology, University of Florida College of Medicine, Gainesville, FL, USA

**Keywords:** Undergraduate medical education, simulation, obstetrics, labour and delivery

## Abstract

**Introducation:**

The aim of this study was to evaluate the impact on knowledge and learner satisfaction of adding a labour and delivery simulator-based training module *versus* a self-study session to the pre-existing theoretical class, in the 5^th^ year undergraduate medical curriculum.

**Methods:**

One hundred and fifty seven students attending the 5-week Obstetrics and Gynecology rotation were enrolled, and 107 completed the study. After a 90-minute “labour and delivery” theoretical interactive class, students were randomized to two groups: the first (n = 56) participated in a 30-minute supervised self-study session, while the second (n = 51) attended a 20–30 minute delivery simulator session. Tests consisting of 10 multiple-choice questions were taken before the theoretical class (pre-test), after the self-study or simulation session (1^st^ post-test) and 12–15 days later (2^nd^ post-test). A subgroup of 53 students participating in this study (27 from the simulation and 26 from the self-study arm) answered six additional questions on satisfaction with the learning experience, at the time of the 1^st^ post-test. Wilcoxon paired rank sum test, Wilcoxon T test, and z-statistic with continuity correction were employed for statistical analysis, setting significance at p < 0.05.

**Results:**

Pre-test scores were similar in both groups (p = 0.9567), but in the first post-test they were significantly higher in the simulation group (p = 0.0017). In the 2^nd^ post-test, scores were again similar in both groups (p = 0.2204). Satisfaction was significantly higher in the simulation group (p < 0.0001).

**Conclusions:**

Adding a simulator-based training session for medical students in management of labour and delivery to the theoretical class led to a higher short-term increase in knowledge and student satisfaction than attending a self-study session. Significant differences in knowledge were no longer demonstrable at 12–15 days.

Lectures and textbooks still form the core of undergraduate teaching in Obstetrics and Gynaecology, but the increased focus on training of practical skills and the limited availability of patients willing to participate in it, has introduced the need for new methodologies. The learning objectives in medical undergraduate training have been extensively reviewed by leading organisations in the field .^1^,^2^ Key recommendations from these documents include implementation of a continuous process of curriculum renewal to adapt to progress in the areas of knowledge, stimulation of student motivation, and use of modern technologies if evidence shows that they are effective.

The term “Best Evidence Medical Education” was coined to describe the implementation of methods and approaches to education based on the best available evidence^3^. This implies the demonstration of benefit in implementing educational tools before making them widely available. Unfortunately, such evidence is available in only a limited number of areas. For instance, problem-based learning has spread rapidly in many medical schools without sound evidence that it leads to greater retention of knowledge, recall of information, or strengthening of hypothetic-deductive reasoning 4.

Medical simulation has recent technology with many potential advantages for undergraduate training, and the idea that medical schools should consider redesigning their curricula in the light of its appearance is gaining wider support. 5–7 Success with the widespread implementation of simulation in medical schools has been reported in selected settings 8, and its use for training students and residents in Obstetrics and Gynaecology has been recommended 9. Simulation has been defined as the “artificial (and almost always simplified) representation of a complex real-world process with sufficient fidelity to achieve a particular goal.”^10^ Its application to medicine was initially limited mostly to Anaesthesiology, Cardiology and Surgery, but is now widespread in other areas, including Obstetrics. It allows learning and practice in a sheltered, protected environment, giving students the chance to feel safe with their performance before moving on to real patients. In undergraduate medical teaching, simulation has the potential to facilitate acquisition of knowledge and skills, to constitute an objective performance assessment tool 11,12, as well as to open up new possibilities for the evaluation process itself .^13^–^16^

Medical students’ cognitive impact using simulation with mannequins has not been widely analysed as a main research outcome. In a recent review of simulation research in the obstetric field, it was noted that very few studies focused on undergraduate medical learning, and all of these evaluated the acquisition and training of procedural skills.^17^ It is not easy to separate the evaluation of the cognitive compartment, when practice learning of a technical skill is being performed, as was amply demonstrated in the United Kingdom multi-center study, evaluating principles for auditing simulated practice learning environments in pre-registration nurses.^18^

Students’ satisfaction is another important aspect of the learning process, as it is strongly related to subsequent motivation. However, evidence on undergraduate medical students’ satisfaction with different methods of clinical education is currently quite limited, due to it's subjectivity and complexity.^19^,^20^ Students’ satisfaction variables have been categorized into three domains^20^ personal (part of the individual's character, such as life satisfaction and self-esteem), interpersonal (relationship between the student and the clinical instructor) and organizational (characteristics that may influence satisfaction such as number of teachers, patients, educational methods, and learned practical skills). Despite the complexity of evaluating this issue, we believe that some degree of feedback on student's satisfaction, such as perception of learning or self-confidence, is important when introducing and evaluating a new educational tool.

In this study, we evaluated the impact on students’ knowledge and satisfaction with the introduction of a labour and delivery simulator-based training module into the existing 5^th^ year undergraduate medical curriculum. Other aspects beyond the cognitive realm, such as technical, non-technical skills, and attitudes were not evaluated. The main research questions underlying this study were: “Do medical students improve their knowledge of the main concepts of labour and delivery, when adding an obstetrical simulator training session to a classical theoretical class?” “Do students feel more satisfied regarding their learning experience and self-confidence, after attending this simulator session?

## Methods

**Figure 1.**
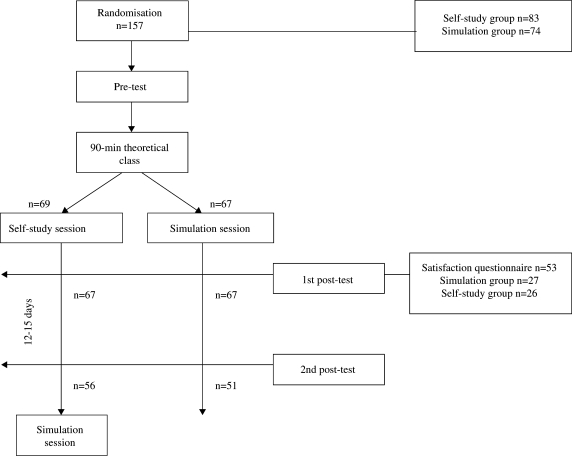
Study flow chart

From September 2004 to April 2006 a total of 157 fifth-year medical students, attending the 5-week Obstetrics and Gynecology rotation (groups of 24 to 26), were invited to participate in the study (Figure 1). The overall objectives and study design were explained to all, and it was underlined that participation was optional, and would not interfere with the subsequent training program or with final grades. All students gave their informed consent to participate.

Students were randomly assigned into two groups using computer-generated random numbers. After a 90-minute “labour and delivery” theoretical interactive class the first group participated in a 30-minute supervised self-study session, where two review chapters were supplied on normal labour and cephalic delivery, with emphasis on the phases of labour, foetal presentations, and cardinal movements of the foetus during delivery. The second group was divided into pairs and attended a 20–30 minute delivery simulator session, using the Noelle^TM^ (Gaumard® Inc. USA) simulator. This full-body, script-driven simulator incorporates a computer controlled mechanical apparatus for fetal descent in a pregnant woman mannequin. The main learning objectives of the session were to evaluate progress of the active phase of labour and to perform the manoeuvres necessary to assist an uneventful cephalic delivery. All students in this group trained vaginal examination for evaluation of cervical characteristics (consistency, dilatation and effacement), diagnosis of an occiput presentation, fetal position and variety. Emphasis was given to proper identification of the sagittal suture, small and large fontanels, and maternal ischial spines for evaluation of fetal descent. For conduction of delivery, students were trained to assess descent of the fetal head, protect the perineum at the time of head delivery, look for nuchal cords after head delivery, deliver the shoulders, and to double-clamp and cut the umbilical cord. After childbirth, students trained the evaluation of placental separation, controlled cord traction, placental delivery and examination, and monitored post-partum uterine contracture.

Knowledge was assessed using a 10-question multiple-choice test, taken before the theoretical class (pre-test), after the self-study or simulation session (1^st^ post-test) and 12–15 days later (2^nd^ post-test). A pool of 30 questions was previously prepared by the faculty member responsible for the theoretical class, and randomly included in the three tests. Test questions focused on the concepts and mechanisms involved during normal labour and cephalic delivery.

Satisfaction was evaluated in a subgroup of 53 students participating in the study, (27 from the simulation arm and 26 from the self-study session), by adding six questions to the 1^st^ post-test. A five-point Likert scale was used (1- totally disagree, 2- disagree, 3- no opinion, 4- agree, 5- totally agree). Four questions centred on students’ perception of learning: “*It consolidated knowledge acquired in the theoretical class”*; “*I learnt something new”*; “*my perception of labour and delivery mechanisms improved”*; “*I was satisfied with the knowledge acquired”*. Questions 5 and 6 centred on learners’ feelings towards the prospect of facing real situations*: “it diminished my anxiety towards future attendance of childbirth”*; “*it increased my confidence with the capacity to assist childbirth”*.

**Statistical Analysis** - Matlab® R2006b was used for statistical analysis. Since the Kolmogorov-Smirnoff test revealed a skewed distribution of scores, non-parametric tests were applied. The Wilcoxon paired rank sum test was used to analyse the evolution of students’ grades in each group. Wilcoxon T test was used to compare grades and their evolution between the groups. Likert scores evaluating satisfaction were grouped into negative (totally disagree + disagree), no opinion, and positive (agree + totally agree). A hypothesis test using the z-statistic, with continuity correction, was used to compare the two independent groups of categorical data. Significance of the tests was set at p < 0.05. Randomisation results were tested for gender, medical course average grades (until the 5^th^ year excluding Obstetrics and Gynecology) and the 5^th^ year theoretical test grade in Obstetrics and Gynecology. Average course grades between included and excluded students were also compared. For validation of the assessment tool, results were correlated with students’ 5^th^-year Obstetrics and Gynecology theoretical test grades, using Spearman's rank correlation. As part of a power analysis, effect sizes for our data were calculated *a posteriori*, according to the procedure described by Siegel^21^, considering a statistical power of 0.95.

## Results

From the total of 157 enrolled students, 50 were excluded after randomization because they did not complete at least one of the scheduled tests (23 from the simulation group and 27 from the self-study session). Thus, only 107 students completed all three tests, 51 from the simulation arm and 56 from the self-study session.

There were no statistical differences between the two study groups regarding gender, medical course average grades, and 5^th^ year Obstetrics and Gynecology theoretical test grades. Also, no statistical difference was detected in medical course average grades, between included and excluded students. Moreover, there was no significant correlation between pre-test results and 5^th^ year Obstetrics and Gynecology theoretical test grades (RHO= -0.078, p = 0.42). On the other hand, a significant correlation was obtained between these grades and 1^st^ and 2^nd^ post-tests results, RHO = 0.22 (p = 0.02) and RHO = 0.59 (p < 0.0001), respectively.

Median scores obtained in the pre-test, 1^st^ and 2^nd^ post-tests are listed in Table 1, together with test score progression, inter-test and inter-group statistical analysis. Pre-test median scores were not significantly different in both groups (p = 0.9567), but 1^st^ post-test median scores and progression between pre-test and 1^st^ post-test were significantly higher in the simulation group (p = 0.0017 and p = 0.0261, respectively). Second post-test mean scores and progression between post-tests were not significantly different in both groups (p = 0.2204 and p = 0.3009, respectively). A positive progression between tests was observed in both groups, reaching statistical significance in all but the simulation group 1^st^ to 2^nd^ post-test progression.

**Table 1.** Simulation and self-study groups test median scores (2.5, 97.5 percentiles) and inter-group* and intra-group** statistical analysis (p).

	Pre-test	1^st^ Post-test	2^nd^ Post-test	Difference Pre-1^st^ post (p)	Difference 1^st^-2^nd^ post (p)
**Simulation (n = 51)**	3 (0, 5)	6 (4, 8)	7 (2.6, 9)	**p < 0.0001**	p = 0.2527
**Self-study (n = 56)**	2.5 (0, 5)	5(2,7.1)	6 (2, 10)	**p < 0.0001**	**p = 0.0170**
**Difference between groups (p)**	p = 0.9567	**p = 0.0017**	p = 0.2204	**p = 0.0261**	p = 0.3009

*Wilcoxon T test

**Wilcoxon paired rank sum test

The effect sizes varied between 0.9 and 1.26. The effect size of the hypothesis test on the 2^nd^ post-test indicates that the statistical test was able to identify differences higher than 12.5%. Therefore, the alternative hypothesis can be stated as “the difference in grades between the two groups is higher than 1.25”.

Students’ self-perception of the learning experience and feelings towards the prospect of facing real situations are listed in Table 2. Overall satisfaction was significantly higher in the simulation group (p < 0.0001).

**Table 2.** Satisfaction evaluation through proportions of a simplified Likert score in the simulation and self-study groups (n = 53), and statistical analysis between groups* (p).

Question	Simplified Likert score	Simulation (n = 27)	Self-study (n = 26)	P
*“I consolidated the knowledge acquired in theoretical class”*	Negative	0	23%	**0.0266**
	No opinion	0	0	
	Positive	100%	77%	**0.0266**

*"I learnt something new"*	Negative	0	23%	**0.0266**
	No opinion	0	0	
	Positive	100%	77%	**0.0266**

*"My perception of labour and delivery mechanisms improved"*	Negative	4%	46%	**0.0011**
	No opinion	0	8%	0.4544
	Positive	96%	46%	**<0.0001**

*"I was satisfied with the knowledge acquired"*	Negative	4%	27%	**0.0481**
	No opinion	0	12%	0.2214
	Positive	96%	61%	**0.0054**

*"It diminished my anxiety towards future attendance of childbirth"*	Negative	19%	46%	**0.0628**
	No opinion	7%	19%	**0.3870**
	Positive	74%	35%	**0.0091**

*“It increased my confidence on the capacity to assist childbirth”*	Negative	11%	65%	**<0.0001**
	No opinion	0	8%	0.4544
	Positive	89%	27%	0,17232

*“Overall satisfaction”*	Negative	6%	38%	**<0.0001**
	No opinion	1%	8%	**0.0113**
	Positive	93%	54%	**<0.0001**

*Hypothesis test using the z-statistic with continuity correction.

## Discussion

In this study, reinforcement of a “labour and delivery” theoretical class with an obstetric simulator-based session led to a significant increase in short-term knowledge, when compared to self-study of similar duration. However, a significant difference in students’ knowledge was no longer demonstrable at 12–15 days. Both groups improved their scores during this period, but the gain only reached statistical significance in the self-study group. This suggests that the control group “caught up” with their counterparts during this period, presumably because they attended other lectures and practical sessions where related subjects were discussed. It is thus possible to hypothesise that faster retention of knowledge can be obtained with simulation training, but that other forms of learning can compensate for this. Another possibility was that the sample size of students attending the 2^nd^ post-test was insufficient to show significant differences in knowledge, or that assessment tools were incapable of detecting these differences. However, the small effect sizes indicate that the sample sizes are adequate to the statistics conducted.

We acknowledge that a 12–15 day period is clearly insufficient to evaluate long-term retention of knowledge. Unfortunately, it was impossible to establish a longer interval, as the faculty felt that students allocated to the self-study arm needed to be compensated with a simulation session, before initiating clinical observation on the third week of rotation. The wide dissemination of simulation technologies, together with the intuitive notion that they are useful and appreciated by students, limits the possibility of withholding them completely from a group of subjects during medical training. A cluster randomized trial design, involving different institutions could overcome this problem, but it would be difficult to control for other aspects of the medical school curriculum influencing results. Therefore, it seems likely that short-term benefit will remain the best obtainable evidence for evaluating the impact on knowledge of labour and delivery simulation technology in undergraduate clinical learning.

A significantly higher satisfaction with the learning experience and increased self-confidence was observed in the simulation group, and this is an important argument in favour of the use of labour and delivery simulators for undergraduate medical teaching, as these aspects can have a profound influence on students’ motivation.

Similar evaluations have been published in other areas of undergraduate medical education. In one study, improved self-confidence of first year medical students was shown, in response to questions on basic physiological principles, after attending a workshop using full-body model-driven simulators.^22^ In a randomized controlled trial evaluating simulation-based training *versus* traditional instruction in critical care and emergency medicine, 38 third-year medical students were evaluated 23. Two written tests were undertaken, just before and after the instruction, in order to evaluate performance. No significant differences were found in results obtained by the two groups, but this could be due solely to the study's small sample size. A recent study evaluated the level of confidence of 33 third-year medical students in performing obstetrical procedures after a “labor and delivery” lecture, followed by training with the Noelle^TM^ simulator, *versus* no further instruction^24^. A self-reported higher level of confidence in performing a vaginal delivery was observed in those who had practiced with the simulator.

Some of the possible limitations of this study involve the absence of *a priori* knowledge of the effect of the intervention in order to calculate an appropriate sample size, and the possible effects of student attrition. The tests used to evaluate student knowledge were limited in strength and time, in order to assure high student participation. However, as already pointed out, the strongest limitation was probably the short duration of follow-up. Learning is a multi-step process involving acquisition, retention and retrieval^25^ and only the short-term implications of this process were evaluated. Similar short-comings are found in the literature involving simulation-based learning. Descriptive studies are more common than outcome-based ones^26^ probably because the opportunities for longitudinal investigation with medical students are limited.^27^

Learning objectives of simulator-based classes (knowledge, skills, and attitudes) need to be clearly addressed when they are introduced in the medical curriculum. For the purpose of this study, the impact of simulation training was restricted to the cognitive aspects or conceptual understanding of the theoretical class. However, it is well known that simulation-based training can promote other aspects of medical education, such as the rapid translation of knowledge (knows) into reasoned action (does).
